# Starting dose and dose adjustment of non-vitamin K antagonist oral anticoagulation agents in a nationwide cohort of patients with atrial fibrillation

**DOI:** 10.1038/s41598-021-99818-4

**Published:** 2021-10-19

**Authors:** L. Gozzo, A. Di Lenarda, F. Mammarella, P. P. Olimpieri, A. Cirilli, M. Cuomo, M. M. Gulizia, F. Colivicchi, G. Murri, S. K. Kunutsor, D. Gabrielli, F. Trotta

**Affiliations:** 1 Agenzia Italiana del Farmaco, Rome, Italy; 2 Cardiovascular Center, University Hospital and Health Services of Trieste, Trieste, Italy; 3 National Reference and High Specialization Hospital “Garibaldi-Nesima” of Catania, Catania, Italy; 4 Cardiology Division, San Filippo Neri Hospital, Rome, Italy; 5 Translational Health Sciences, Bristol Medical School, University of Bristol, Bristol, UK; 6 Cardiology Division, Hospital “Murri”, Fermo, Italy; 7 Heart Care Foundation, Florence, Italy

**Keywords:** Atrial fibrillation, Drug therapy

## Abstract

This study aims to provide real-world data about starting-dose of NOACs and dose-adjustment in patients with atrial fibrillation (AF). In fact, even if new oral anticoagulation agents (NOACs) have a predictable effect without need for regular monitoring, dose-adjustments should be performed according to the summary of product information and international guidelines. We employed the Italian Medicines Agency monitoring registries comprising data on a nationwide cohort of patients with AF treated with NOACs from 2013 to 2018. Logistic regression analysis was used to evaluate the determinants of dosage choice. During the reference period, treatment was commenced for 866,539 patients. Forty-five percent of the first prescriptions were dispensed at a reduced dose (dabigatran 60.3%, edoxaban 45.2%, apixaban 40.9%, rivaroxaban 37.4%). The prescription of reduced dose was associated with older age, renal disease, bleeding risk and the concomitant use of drugs predisposing to bleeding, but not with CHA_2_DS_2_-VASc and HAS-BLED. A relative reduction of the proportion of patients treated with low dosages was evident overtime for dabigatran and rivaroxaban; whereas prescription of low dose apixaban and edoxaban increased progressively among elderly patients. Evidence based on real-world data shows a high frequency of low dose prescriptions of NOACs in AF patients. Except for older age, renal disease, bleeding risk and the concomitant use of drugs predisposing to bleeding, other factors that may determine the choice of reduced dose could not be ascertained. There may be potential under-treatment of AF patients, but further evaluation is warranted.

## Introduction

Atrial fibrillation (AF) is the most common cardiac arrhythmia associated with an increased risk of stroke and thromboembolism, which can be prevented with the use of anticoagulants^1^ such as Vitamin K antagonists (VKAs) or non-vitamin K antagonist oral anticoagulation (NOAC) agents. NOACs have less treatment-related issues compared to VKAs^2^; however, dose-adjustment at first prescription and re-evaluation of patients is necessary in order to assess the appropriateness of treatment dosage^1,3^.

Prescription of anticoagulants should be made according to regulatory authority requirements, evidence-based guidelines and patient characteristics; furthermore, the appropriate choice of dosages should also take into account the benefit-to-harm ratio of under or overdose.

In general, dose adjustments should be considered in elderly patients, in those with moderate/severe renal dysfunction, low body weight, elevated risk of bleeding and use of concomitant drugs, which may impact on P-glycoprotein (P-gp) transport system and/or hepatic cytochrome p-450^3,4^. More specifically, renal function defines the choice of dose and contraindications for all NOACs, considering that their elimination is largely dependent on kidneys and patients with end-stage renal disease were excluded from pivotal clinical trials^5^. Each NOAC is available at different recommended doses for stroke prevention in patients with AF^3^. Specific recommendations should be considered for each drug to identify the right treatment schedule, avoiding dosing errors which still represent important practical issues in the management of patients treated with NOACs^3^.

Using a large nationwide cohort of patients with AF enrolled in the Italian Medicines Agency (AIFA) database, the aims of this study were to evaluate the characteristics and prevalence of patients treated with reduced dose of NOACs, to evaluate the determinants of dosage choice and to analyze the proportion of dose reductions that might be considered appropriate, based on European Guideline recommendations^1,3^.

## Materials and methods

Our nationwide cohort includes all Italian patients with AF who were prescribed a NOAC through the AIFA web-based monitoring registry between June 2013 and December 2018. In Italy, it is mandatory to perform NOAC prescription through the AIFA web-based monitoring registry, which collects all clinical and treatment information required for NOAC reimbursement by the Italian NHS^6^. According to the Italian laws, this obligation does not require any informed consent or ethical committee approval. (Decree 196/2003 “Italian Privacy Code”; Decree 101/2018 “Harmonization Decree" harmonizing the Italian data protection laws with the provision of the General Data Protection Regulation 679/2016—GDPR), Law 135/2012; Law 125/2015). Anyhow, the physicians are required to inform the patient about the purposes of the monitoring, upon inclusion in the system.

Baseline characteristics and proportion of patients on reduced and standard dose were evaluated for all NOACs as well as subgroups of patients with potential indication for dose reduction. We focused our analysis on dose at the first prescription and dose adjustments during the first 3 years of observation.

Criteria for prescription of a reduced dose are different for each NOAC^1,3^*,* as defined in the summary of product characteristics (SmPC). The reduced dose of dabigatran is recommended for patients aged ≥ 80 years or in case of concomitant treatment with verapamil; moreover, dose of dabigatran should be selected based on the individual thromboembolic and bleeding risk. The reduced dose of rivaroxaban is indicated if Creatinine Clearance (CrCl) < 50 mL/min. Low dose of apixaban is prescribed in the presence of two out of three criteria: weight ≤ 60 kg, age ≥ 80 years and serum creatinine ≥ 133 mmol/L (1.5 mg/dL). For edoxaban, low dose is prescribed if weight ≤ 60 kg, CrCl ≤ 50 mL/min or there is concomitant therapy with strong P-Gp inhibitor. Since not all data on these criteria were available in the AIFA Registry, we limited our analyses to evaluate the potential appropriateness of dose prescription to age, normal/impaired renal and liver function, previous major bleeding or bleeding predisposition and medications predisposing to bleeding. Specifically, the presence of impaired renal function was used to analyze the appropriateness of dose-reduction of rivaroxaban and edoxaban, and age ≥ 80 years AND impaired renal function for apixaban. Finally, we considered appropriate criteria for a reduced dose prescription of dabigatran: age ≥ 80 years OR HASBLED ≥ 3^7^.

Continuous variables were expressed as mean and min to max range. Categorical variables were reported as numbers and percentages. Determinants for selecting a reduced dose were assessed using a multivariate logistic regression performed with SAS 9.4. Date of prescription was also taken into account to highlight how the clinician preference for a reduced dose changed overtime. Data were analyzed with the statistical software R^8^ using the ggplot2 package for data visualization^9^.

## Results

From June 2013 to December 2018, the AIFA database collected data for 927523 treatments initiated with a NOAC, corresponding to 866539 patients with AF. The discrepancy between the number of treatments and the number of patients is due to switches among NOACs. Rivaroxaban was the most prescribed NOAC among the four drugs (33.2%), followed by apixaban (30.8%), dabigatran (26.3%) and edoxaban (9.7%), which was the last one approved. More than half of the first prescriptions were performed at the approved standard dose(Table 1), while the remaining percentage was for reduced doses of NOACs. Dabigatran was the drug with the highest rate of prescriptions at the reduced dose (60.3%) (Tables S1a and S1b).

**Table 1:** Baseline characteristics of the population according divided according to dose of NOACs at first prescription in AIFA Registry.

	Standard dose	Reduced dose	Overall
N° treatments	507,548 (54.72%)	419,975 (45.28%)	927,523 (100.00%)
**Sex**			
Female	223,266 (43.99%)	239,511 (57.03%)	462,777 (49.89%)
Male	284,282 (56.01%)	180,464 (42.97%)	464,746 (50.11%)
**Median age (range)**	74 (18–102)	83 (18–109)	78 (18–109)
Age < 65	87,692 (17.28%)	9821 (2.34%)	97,513 (10.51%)
Age ≥ 65 and < 75	190,581 (37.55%)	45,943 (10.94%)	236,524 (25.5%)
Age ≥ 75 and < 85	194,718 (38.36%)	209,268 (49.83%)	403,986 (43.56%)
Age ≥ 85	34,557 (6.81%)	154,943 (36.89%)	189,500 (20.43%)
CHA_2_DS_2_-VASc Score 0	5791 (1.14%)	442 (0.11%)	6233 (0.67%)
CHA_2_DS_2_-VASc Score 1	33,827 (6.66%)	2954 (0.7%)	36,781 (3.97%)
CHA_2_DS_2_-VASc Score 2	87,059 (17.15%)	18,062 (4.3%)	105,121 (11.33%)
CHA_2_DS_2_-VASc Score 3	131,845 (25.98%)	70,404 (16.76%)	202,249 (21.81%)
CHA_2_DS_2_-VASc Score 4	124,502 (24.53%)	131,198 (31.24%)	255,700 (27.57%)
CHA_2_DS_2_-VASc Score 5	71,059 (14%)	101,016 (24.05%)	172,075 (18.55%)
CHA_2_DS_2_-VASc Score 6 +	53,465 (10.53%)	95,899 (22.83%)	149,364 (16.1%)
HAS-BLED Score 0	16,554 (3.26%)	1356 (0.32%)	17,910 (1.93%)
HAS-BLED Score 1	93,919 (18.5%)	39,652 (9.44%)	133,571 (14.4%)
HAS-BLED Score 2	212,818 (41.93%)	165,812 (39.48%)	378,630 (40.82%)
HAS-BLED Score 3	123,757 (24.38%)	128,434 (30.58%)	252,191 (27.19%)
HAS-BLED Score 4 +	60,500 (11.92%)	84,721 (20.17%)	145,221 (15.66%)
Diabetes history	98,032 (19.31%)	84,054 (20.01%)	182,086 (19.63%)
Hypertension history	434,300 (85.57%)	365,327 (86.99%)	799,627 (86.21%)
Stroke/TIA/Thromboembolism history	79,712 (15.71%)	82,912 (19.74%)	162,624 (17.53%)
Vascular disease history	122,184 (24.07%)	128,483 (30.59%)	250,667 (27.03%)
CHF history	115,797 (22.81%)	148,397 (35.33%)	264,194 (28.48%)
Alcohol use	28,231 (5.56%)	17,525 (4.17%)	45,756 (4.93%)
Liver disease	3889 (0.77%)	4880 (1.16%)	8769 (0.95%)
Renal^a^ disease	8122 (1.6%)	42,920 (10.22%)	51,042 (5.5%)
Prior major bleeding or predisposition to bleeding	41,317 (8.14%)	61,880 (14.73%)	103,197 (11.13%)
Labile INR	108,349 (21.35%)	87,797 (20.91%)	196,146 (21.15%)
Prior anticoagulant treatment (AVK)	152,638 (30.07%)	119,824 (28.53%)	272,462 (29.38%)
Medication usage predisposing to bleeding	73,221 (14.43%)	80,020 (19.05%)	153,241 (16.52%)
Prior NOAC treatment (switch)	28,520 (5.62%)	32,210 (7.67%)	60,730 (6.55%)

Data are expressed as mean and min–max range or numbers and percentages.

^a^Renal disease = renal transplantation or dialysis or plasma creatinin > 200 µmol/L.

### Characteristics of the cohort according to dose

Table 1 shows the baseline characteristics of the cohort divided according to dose at first prescription. The median age was 83 years (range 18–109) in the reduced dose and 74 years (range 18–102) in the standard dose group, with 36.9% and 6.8% of patients aged 85 and older, respectively. Proportion of females was higher in the reduced dose group (57.1%) compared to the standard dose one (44.0%). Clinical characteristics most prevalent in the reduced dose group were congestive heart failure and renal disease (35.3% versus 22.8% and 10.2% versus 1.6%, in the standard dose group, respectively), followed by a past medical history of major bleeding or bleeding predisposition (14.7% vs 8.1%) and the concomitant use of medications predisposing to bleeding (19.1% vs 14.4%).

### Factors associated with dosage choice

**Figure 1:**
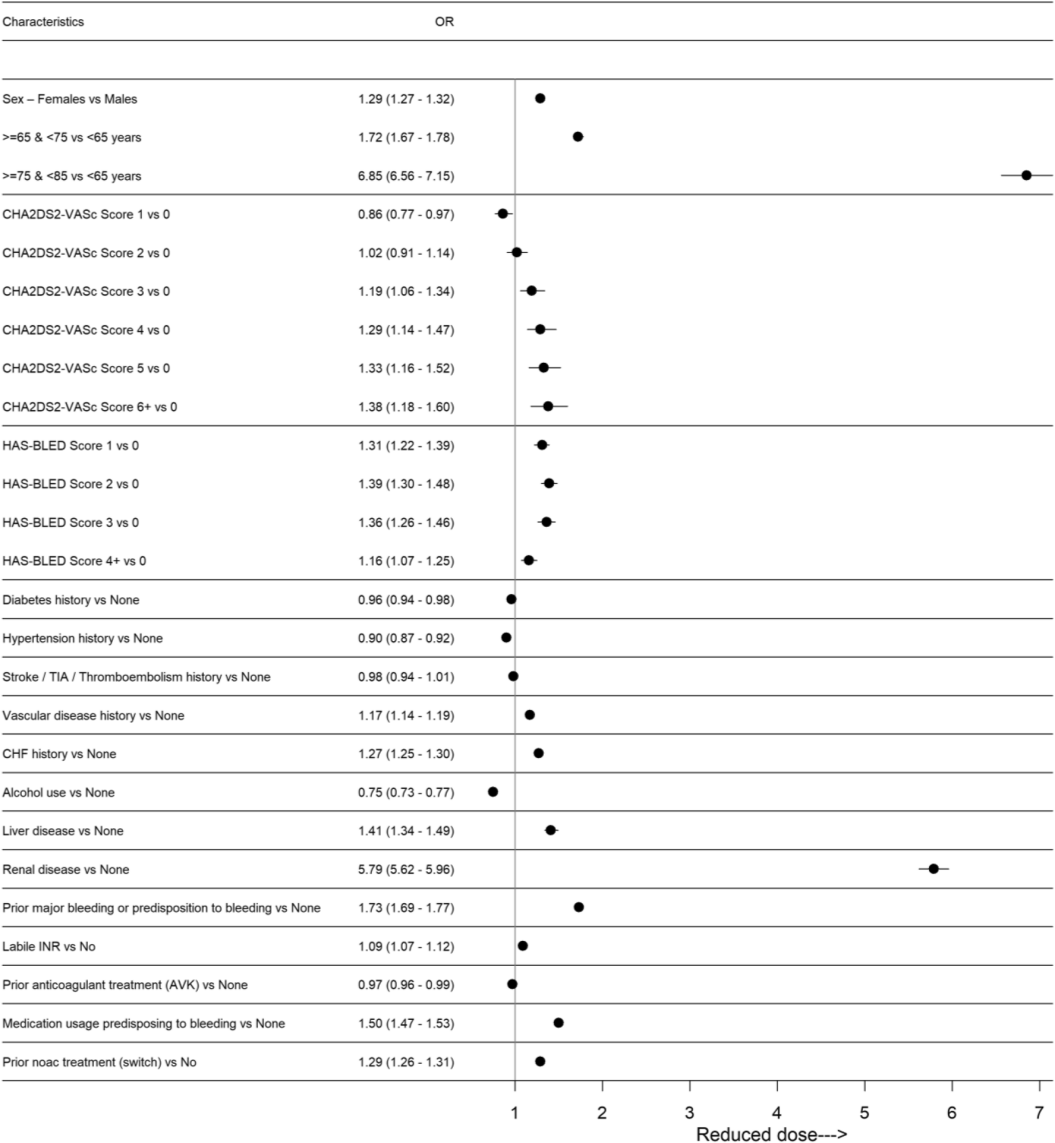
Association between baseline characteristics and choice of reduced dose of NOACs at first prescription in 866,539 patients enrolled in AIFA Registry (2013–2018). Data are expressed as odds ratio and confidence intervals for the baseline characteristics. For visual purposes, the odds ratio associated for age class 85 + (OR 27.22 26.03–28.46, reference < 65 years old) is not shown. Plot has been made using the statistical software R^8^ and the forestplot package^10^.

**Figure 2:**
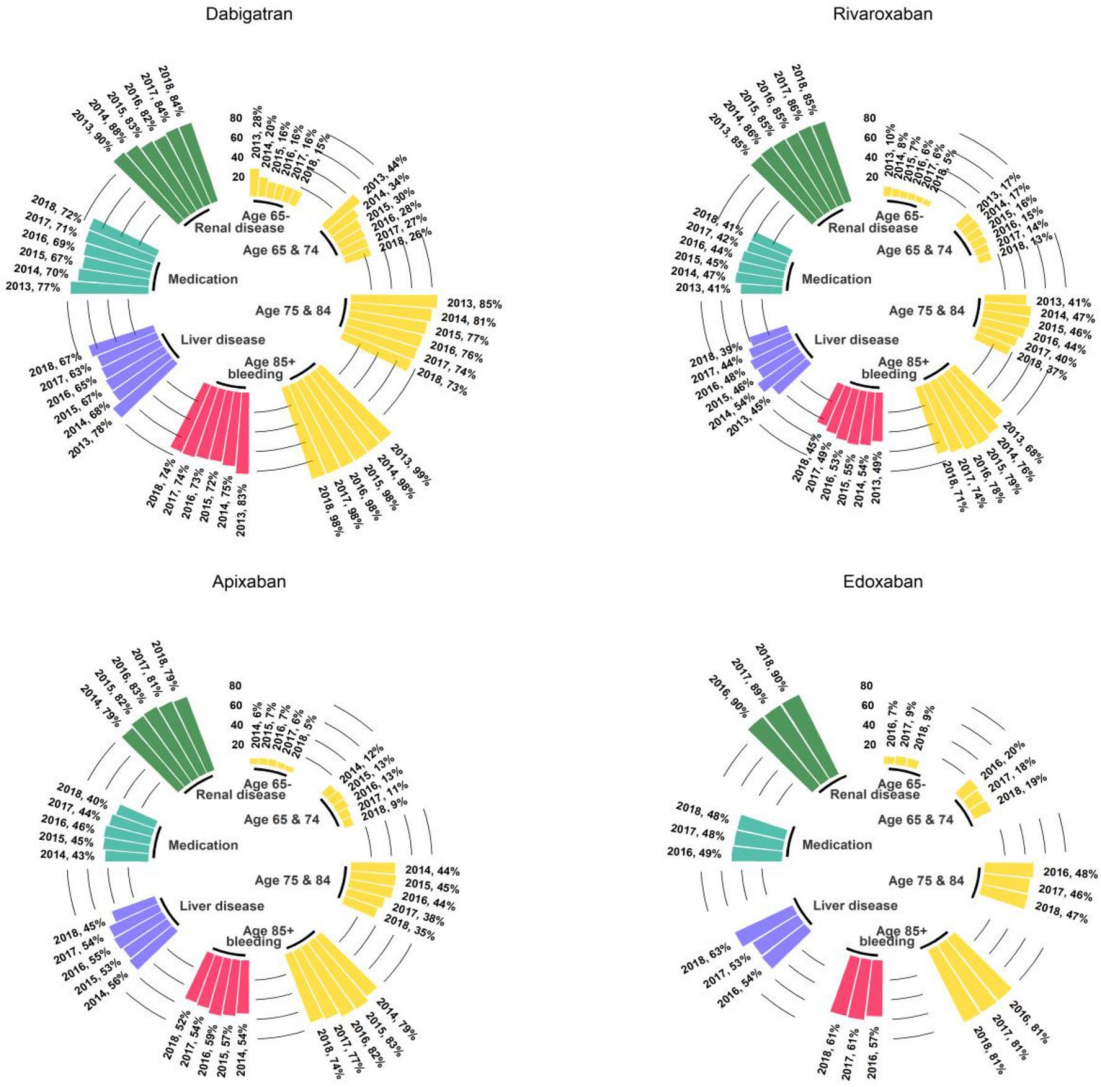
Percentage of low dose at first prescription from 2013 to 2018 for all NOACs separately according to the main clinical information available in the Registry. Plot has been made using the statistical software R^8^ the ggplot2 package^10^.

Figure 1 and Table 2 show the associations between clinical characteristics at baseline and the choice of reduced dose at first prescription. We found the strongest association with age (OR 27.2, 95% CI 6.0–28.5 and 6.9, 95% CI 6.6–7.2 for age group ≥ 85 and 75–84 respectively compared to the < 65-year group). More than 80% of patients aged 85 or older were treated with a reduced dose, with substantial differences depending on the drug choice. Almost all older patients in the dabigatran group (98%) were initiated with the adjusted dose, and about 80% of all patients treated with other NOACs (Fig. 2). There was a significant association of reduced dose at first prescription with renal disease (OR 5.8, 95% CI 5.6–6.0), which was more evident for edoxaban (OR 11.8, 95% CI 10.7–13.1) and rivaroxaban (OR 9.9, 95% CI 9.3– 10.4). Both bleeding risk and the concomitant use of drugs predisposing to bleeding were each associated with reduced dose at first prescription for all NOACs, but the associations were more evident for dabigatran (OR 2.2, 95% CI 2.1–2.3 and OR 2.1, 95% CI 2.0–2.2 respectively). Neither the CHA_2_DS_2_-VASc nor the HAS-BLED scores were associated with reduced dose at first prescription.

**Table 2:** Association between baseline characteristics and choice of reduced dose at first prescription in 927,523 NOAC treatments in AIFA Registry (2013–2018).

	OR (95% CI) Dabigatran	OR (95% CI) Rivaroxaban	OR (95% CI) Apixaban	OR (95% CI) Edoxaban
**Sex—Females vs Males**	1.25 (1.19–1.31)	1.25 (1.21–1.29)	1.47 (1.42–1.52)	1.82 (1.7–1.95)
≥ 65 and < 75 vs < 65 years	1.8 (1.69–1.91)	1.74 (1.63–1.85)	1.57 (1.46–1.7)	2.05 (1.82–2.32)
≥ 75 and < 85 vs < 65 years	12.42 (11.32–13.63)	6.29 (5.81–6.82)	7.42 (6.78–8.12)	6.94 (5.91–8.14)
≥ 85 vs < 65 years	180.41 (160.24–203.13)	24.82 (22.88–26.93)	38.2 (34.86–41.86)	32.78 (27.88–38.55)
CHA_2_DS_2_-VASc Score1 vs 0	0.74 (0.63–0.87)	1.22 (0.94–1.59)	0.63 (0.47–0.85)	1.21 (0.75–1.93)
CHA_2_DS_2_-VASc Score2 vs 0	0.82 (0.69–0.98)	1.73 (1.33–2.26)	0.64 (0.48–0.87)	1.37 (0.85–2.22)
CHA_2_DS_2_-VASc Score 3 vs 0	0.96 (0.79–1.16)	2.16 (1.64–2.84)	0.72 (0.53–0.98)	1.58 (0.96–2.61)
CHA_2_DS_2_-VASc Score 4 vs 0	1.07 (0.85–1.33)	2.45 (1.84–3.25)	0.8 (0.58–1.1)	1.71 (1.01–2.89)
CHA_2_DS_2_-VASc Score 5 vs 0	1.14 (0.89–1.47)	2.56 (1.9–3.45)	0.81 (0.58–1.12)	1.76 (1.01–3.07)
CHA_2_DS_2_-VASc Score 6 + vs 0	1.34 (0.99–1.82)	2.69 (1.96–3.69)	0.84 (0.59–1.19)	1.84 (1.01–3.37)
HAS-BLED Score1 vs 0	1.28 (1.16–1.4)	1.58 (1.37–1.82)	1.56 (1.3–1.87)	0.87 (0.72–1.07)
HAS-BLED Score 2 vs 0	1.38 (1.25–1.52)	1.77 (1.52–2.05)	1.9 (1.58–2.28)	0.91 (0.74–1.12)
HAS-BLED Score 3 vs 0	1.32 (1.18–1.47)	1.83 (1.57–2.14)	2 (1.66–2.42)	0.84 (0.67–1.05)
HAS-BLED Score 4 + vs 0	1.04 (0.91–1.18)	1.7 (1.44–2.01)	1.91 (1.56–2.33)	0.69 (0.54–0.9)
Diabetes history vs None	0.92 (0.88–0.96)	0.98 (0.95–1.01)	0.95 (0.92–0.99)	0.94 (0.88–1.01)
Hypertension history vs None	0.88 (0.83–0.93)	0.88 (0.84–0.92)	0.74 (0.71–0.78)	0.81 (0.75–0.88)
Stroke/TIA/Thromboembolism history vs None	0.85 (0.78–0.93)	0.89 (0.84–0.95)	0.88 (0.83–0.94)	1 (0.88–1.14)
Vascular disease history vs None	1.29 (1.23–1.35)	1.13 (1.09–1.17)	1.2 (1.16–1.24)	1.13 (1.06–1.22)
CHF history vs None	1.18 (1.12–1.24)	1.35 (1.31–1.4)	1.45 (1.4–1.49)	1.4 (1.31–1.5)
Alcohol use vs None	0.74 (0.7–0.78)	0.69 (0.65–0.72)	0.61 (0.58–0.64)	0.8 (0.73–0.87)
Liver disease vs None	1.57 (1.41–1.74)	1.06 (0.96–1.17)	1.35 (1.23–1.49)	1.48 (1.22–1.78)
Renal disease^a^ vs None	3.23 (2.98–3.51)	9.86 (9.33–10.41)	6.26 (5.97–6.56)	11.81 (10.66–13.08)
Prior major bleeding or predisposition to bleeding vs None	2.22 (2.12–2.33)	1.53 (1.47–1.59)	1.75 (1.69–1.82)	1.81 (1.68–1.94)
Labile INR vs No	1.17 (1.12–1.22)	1.04 (0.99–1.08)	1.13 (1.08–1.19)	1.1 (1–1.2)
Prior anticoagulant treatment (AVK) vs None	1.01 (0.98–1.04)	0.83 (0.8–0.85)	0.73 (0.7–0.75)	0.8 (0.75–0.85)
Medication usage predisposing to bleeding vs None	2.09 (2.01–2.18)	1.28 (1.24–1.33)	1.14 (1.1–1.19)	1.24 (1.15–1.32)
Prior NOAC treatment (switch) vs None	1.52 (1.42–1.62)	1.6 (1.55–1.66)	1.59 (1.54–1.64)	1.98 (1.88–2.08)

Data are expressed as odds ratio and confidence intervals for the baseline characteristics for each NOAC.

^a^Renal disease = renal transplantation or dialysis or plasma creatinin > 200 µmol/L.

### Trend of NOAC dose prescriptions

**Figure 3:**
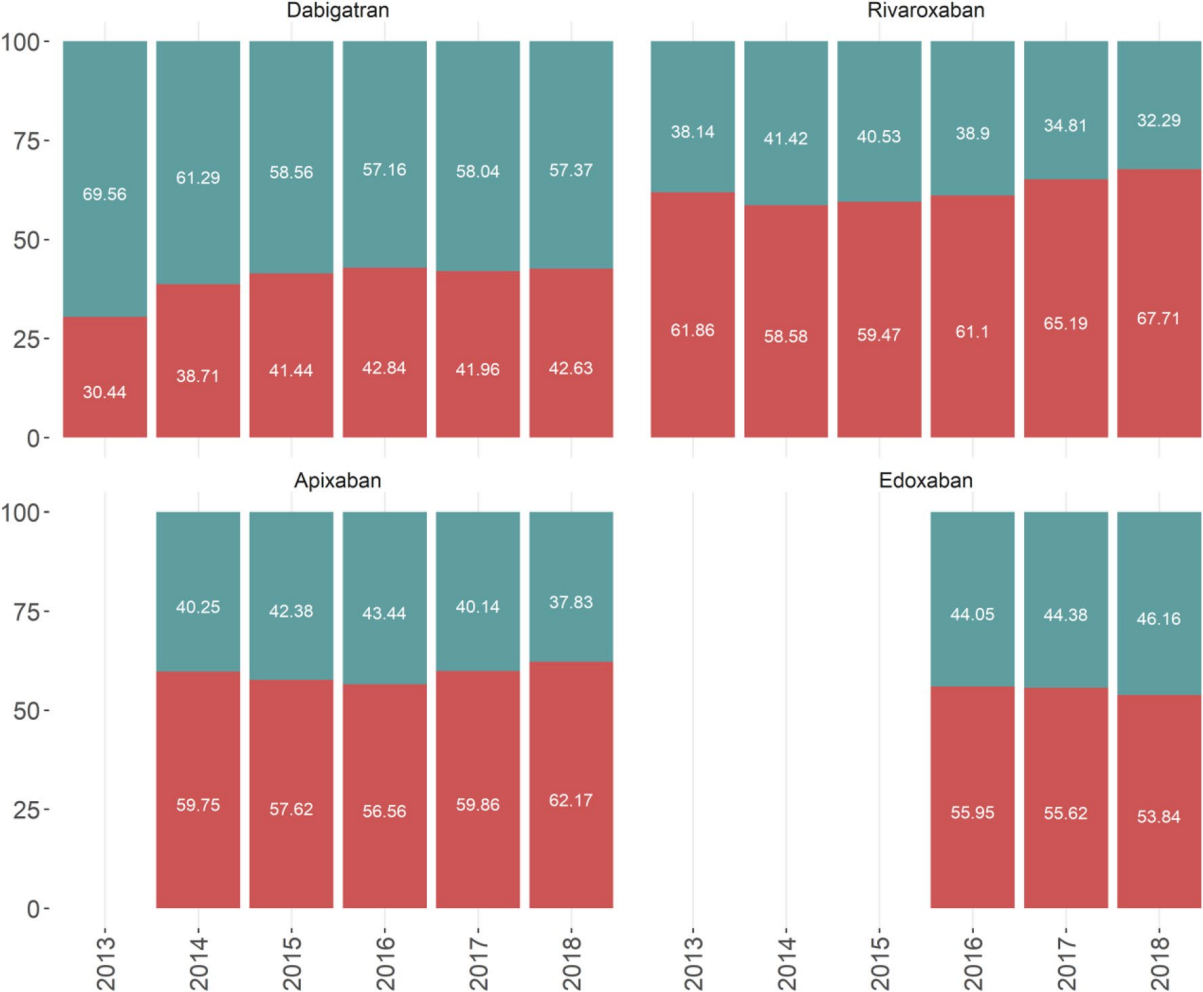
Differences in proportions of dosage choice among 927,523 NOAC treatments based on year of first prescription (2013–2018) in AIFA Registry. Red bars refer to proportion of patients on standard dose of NOACs; green bars refer to adjusted dose of NOACs. Plot has been made using the statistical software R^8^ the ggplot2 package^10^.

Generally, we observed a relative reduction of the number of patients treated with low dosages (from 48.3% in 2014 to 41.5% in 2018), with differences for each drug based on year of first prescription (Fig. 3). For example, patients treated with an adjusted initial dose of dabigatran were approximately 70% in 2013 and 61% in 2014 and decreased progressivelyto 57% in 2018. However, for rivaroxaban, proportion of patients prescribed reduced doses decreased gradually from 2016 to 2018.

The overall trend of reduced dose prescriptions remained relatively stable for apixaban and edoxaban, with only two full years of observation for the latter. In apixaban patients this was explained by a decrease in the frequency of low dose prescriptions for people aged 85 or more (from 79% in 2014 to 74% in 2018) (Fig. 2), associated with a progressive increase in treated people who were aged 85 or more (from 20.9% in 2014 to 27.5% in 2018) (Table S2). Overall, we observed a relative reduction of the number of patients treated with adjusted dosages across all age classes and in those at risk of bleeding and with renal disease (Fig. 2). This trend was in parallel to a general reduction overtime of the proportion of treated patients at higher risk for all NOACs, in particular those with renal and hepatic comorbidities, prior major bleeding or predisposition to bleeding (Table S2).

Finally, marked changes in the population treated with reduced dose of rivaroxaban were observed for all clinical variables, with the exception of renal disease. The 12,000 patients enrolled to undergo cardioversion on rivaroxaban (the only NOAC with specific indication in Italy since 2016) were significantly younger (median age 68 years) and with less co-morbidities compared to the rest of the population (data not shown).

### Dose-adjustment during follow-up

More than half of treatments (485,057, 52.3%) were maintained after 12 months from first prescription. Among these patients, 277,727 (57.3%) were prescribed standard dose, 207,330 (42.3%) on reduced dose. Dose-adjustment was prescribed in 31,171 renewals (6.4%; Table 3), more frequently as a reduction of standard doses compared to the increase of reduced doses (8.1% versus 4.2%). In accordance with SmPC, the most important factors associated with dose-reduction were older age (OR 5.0, 4.4–5.6 and 11.8, 10.4–13.4 for age group 75–84 and ≥ 85, respectively) and renal disease (OR 1.7, 1.6–1.9). The drug with the highest odds of dose-reduction at the second prescription was dabigatran (OR 2.0, 2.0–2.1).

**Table 3:** Factors associated to the switch from standard to reduced dose and from reduced to standard dose in 31,171 s prescriptions.

	Switch from standard to reduced dose OR (95% CI)	Switch from reduced to standard dose OR (95% CI)
Sex—Females vs Males	1.18 (1.11–1.24)	0.77 (0.71–0.84)
NOAC (ref Apixaban) Edoxaban vs Apixaban	1.1 (1.03–1.17)	0.54 (0.49–0.61)
NOAC (ref Apixaban) Dabigatran vs Apixaban	2.04 (1.96–2.13)	0.26 (0.24–0.27)
NOAC (ref Apixaban) Rivaroxaban vs Apixaban	1.16 (1.12–1.2)	0.6 (0.57–0.63)
≥ 65 and < 75 vs < 65 years	1.76 (1.6–1.93)	0.54 (0.47–0.61)
≥ 75 and < 85 vs < 65 years	4.95 (4.38–5.6)	0.18 (0.15–0.22)
≥ 85 vs < 65 years	11.82 (10.4–13.43)	0.07 (0.06–0.08)
CHA_2_DS_2_-VASc Score1 vs 0	1.03 (0.72–1.49)	0.79 (0.53–1.18)
CHA_2_DS_2_-VASc Score2 vs 0	1.02 (0.7–1.47)	0.69 (0.45–1.04)
CHA_2_DS_2_-VASc Score 3 vs 0	1.17 (0.79–1.72)	0.64 (0.41–0.99)
CHA_2_DS_2_-VASc Score 4 vs 0	1.3 (0.87–1.95)	0.6 (0.37–0.97)
CHA_2_DS_2_-VASc Score 5 vs 0	1.33 (0.86–2.03)	0.57 (0.33–0.98)
CHA_2_DS_2_-VASc Score 6 + vs 0	1.37 (0.86–2.18)	0.55 (0.3–1.02)
HAS-BLED Score1 vs 0	1.15 (0.96–1.37)	0.97 (0.75–1.26)
HAS-BLED Score 2 vs 0	1.2 (1–1.44)	0.84 (0.64–1.1)
HAS-BLED Score 3 vs 0	1.18 (0.97–1.44)	0.79 (0.59–1.05)
HAS-BLED Score 4 + vs 0	1.2 (0.96–1.49)	0.7 (0.51–0.97)
Diabetes history vs None	1.11 (1.05–1.18)	1.05 (0.96–1.15)
Hypertension history vs None	0.94 (0.88–1.01)	1.13 (1.01–1.26)
Stroke/TIA/Thromboembolism history vs None	0.98 (0.89–1.09)	1.07 (0.91–1.25)
Vascular disease history vs None	1.13 (1.07–1.19)	1.06 (0.97–1.15)
CHF history vs None	1.4 (1.32–1.48)	0.92 (0.85–1.01)
Alcohol use vs None	0.86 (0.8–0.93)	1.37 (1.22–1.53)
Liver disease vs None	1.06 (0.91–1.25)	0.92 (0.74–1.13)
Renal disease^a^ vs None	1.71 (1.55–1.89)	0.79 (0.71–0.87)
Prior major bleeding or predisposition to bleeding vs None	1.16 (1.09–1.23)	0.96 (0.89–1.05)
Labile INR vs No	1.03 (0.97–1.1)	1.01 (0.91–1.12)
Prior anticoagulant treatment (AVK) vs None	0.94 (0.9–0.99)	1.03 (0.95–1.11)
Medication usage predisposing to bleeding vs None	1.12 (1.06–1.18)	1.33 (1.23–1.44)
Prior NOAC treatment (switch) vs No	1.04 (0.97–1.11)	0.85 (0.78–0.93)

Data are expressed as odds ratio and confidence intervals for the baseline characteristics and previous NOAC treatment.

^a^Renal disease = renal transplantation or dialysis or plasma creatinin > 200 µmol/L.

On the other hand, the increase to standard dose was associated with alcohol use (OR 1.4, 1.2–1.5) and medication predisposing to bleeding, mainly antiplatelets (OR 1.3, 1.2–1.4).

The number of patients still on treatment after 36 months from the first prescription was 143,392 (15.5%) of which 37.4% were treated with dabigatran, 35.4% with rivaroxaban and 27.2% with apixaban. No patients treated with edoxaban had a third renewal at the time of the data analysis.

Among them, 21,891 (15.3%) had at least one dose-adjustment during their treatment. Overall, the AIFA registries collected 22,443 dosage switches (Table 4).

**Table 4:** Absolute and relative frequencies of dosage adjustments from standard to reduced dose and from reduced to standard dose at first and subsequent prescriptions.

	12 months N° (%)	24 months N° (%)	36 months N° (%)	Overall
**Switches form standard to reduced dose**				
Dabigatran (53,689)	1691 (3.15%)	1604 (2.99%)	1567 (2.92%)	4862 (9.06%)
Rivaroxaban (50,737)	2291 (4.52%)	2259 (4.45%)	2144 (4.23%)	6694 (13.19%)
Apixaban (38,966)	1662 (4.27%)	1536 (3.94%)	1422 (3.65%)	4620 (11.86%)
**Switches form reduced to standard dose**				
Dabigatran (53,689)	805 (1.5%)	660 (1.23%)	646 (1.2%)	2111 (3.93%)
Rivaroxaban (50,737)	614 (1.21%)	621 (1.22%)	722 (1.42%)	1957 (3.86%)
Apixaban (38,966)	707 (1.81%)	761 (1.95%)	731 (1.88%)	2199 (5.64%)

### Appropriateness of adjusted dose prescription of NOACS

**Figure 4:**
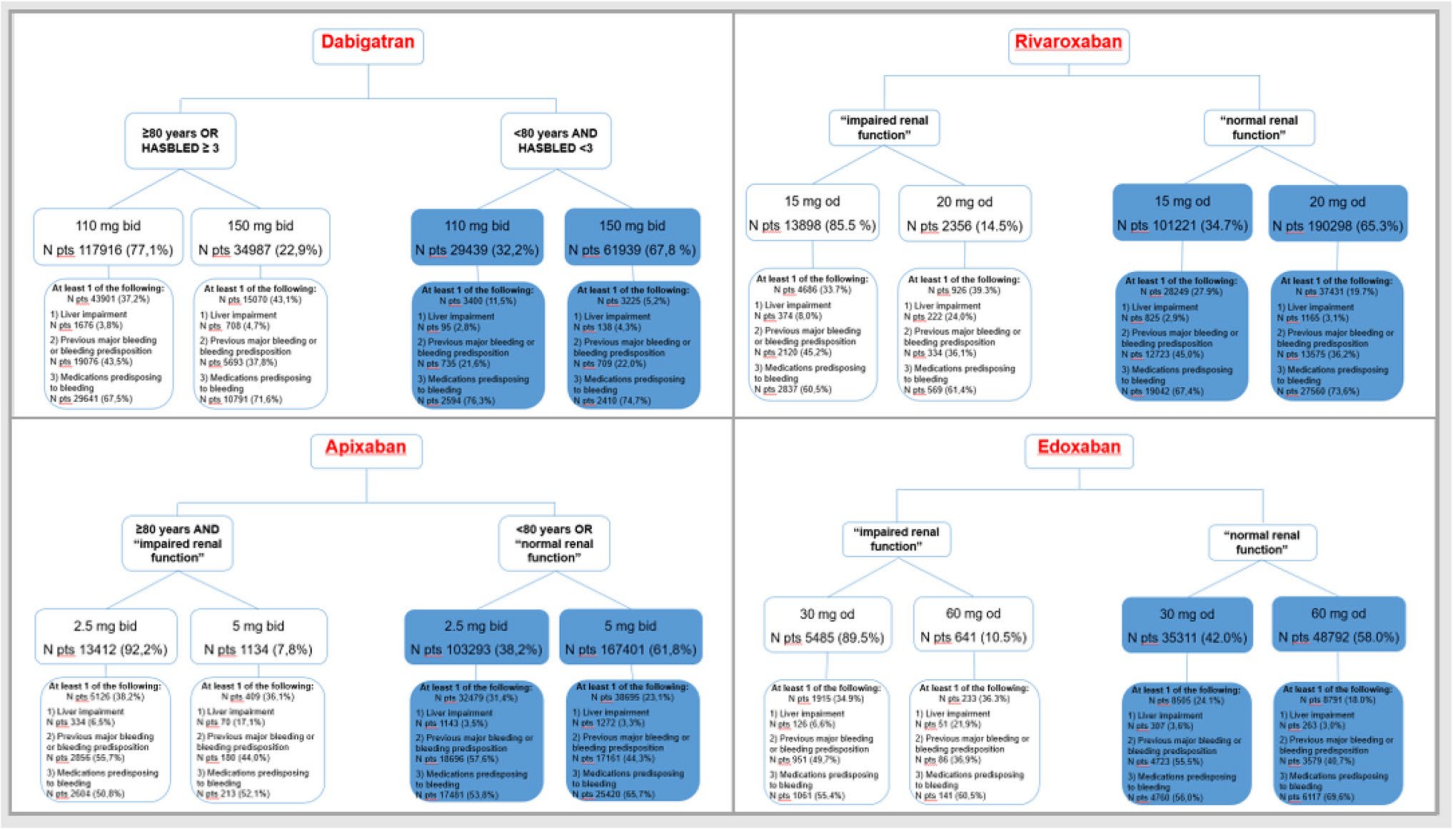
Proportion of patients on standard or adjusted dose on the base of the baseline characteristics potentially influencing the dose choice among 927,523 NOAC treatments in AIFA Registry.

For each drug we analyzed the proportion of patients on standard or adjusted dose on the basis of their specific characteristics potentially influencing the choice (Fig. 4).

Thirty-two percent of patients on dabigatran were potentially undertreated, being < 80 years and with HASBLED < 3. Among these patients, only 11% showed some predisposition to bleeding according to clinical history or associated medications. On the other hand, 22% of patients ≥ 80 years or at high risk of bleeding were potentially over-treated.

The choice of rivaroxaban and edoxaban dose was mainly based on renal function. Among patients without impaired renal function certified by prescribing physician, 34% of those on rivaroxaban, and 42% of those on edoxaban were treated with the reduced dose. These drugs were potentially under-dosed since only about one fourth of them had some characteristics predisposing to bleeding. However, since the AIFA Registry allows to identify only severe renal impairment (renal transplantation or dialysis or plasma creatinin > 200 µmol/L), the group with normal renal function could include some patients with renal diseases, thus justifying the high prevalence of dose reduction.

On the contrary, a relative minority (10 to 15%) of patients, classified as having impaired renal function, was on full dose of NOAC treatment, thus potentially overdosed.

Finally, 38% of patients younger than 80 or with normal renal function were on reduced dose of apixaban. Among these patients, only 31% had a predisposition to bleeding. Therefore, a large population was potentially undertreated. On the other hand, 92% of elderly patients (≥ 80 years) with impaired renal function were on low dose of apixaban.

## Discussion

In the present analysis we described the contemporary practice of NOAC dose-adjustments, and its appropriateness based on European Guideline recommendations^1,3^ in a large (almost 1 million) nationwide community of patients with AF enrolled in the AIFA Registry^6^.

Multiple international randomized trials demonstrated overall improved outcomes in patients receiving pre-specified and tested NOAC doses compared with those for warfarin^11–15^. The implementation of these therapies in appropriate patients at the appropriate doses, consistent with clinical guidelines and drugs labeling, is paramount to reduce the stroke burden in AF, while a relative increase in risk of bleeding cannot be ruled out^16^.

The criteria to adjust the NOAC dose are different and may be interpreted individually for each patient treated with a specific molecule.

Two types of dose-reduction can be performed, the so-called “horizontal” one and the “vertical” one. The former is applicable only for dabigatran, it is independent from patient characteristics and totally at the physician’s discretion^17^. On the contrary, “vertical” dose-reduction is mandatory according to specific patient characteristics^14,17^.

In our study, almost half of first prescriptions were performed at the reduced dose of NOACs, with dabigatran having the highest rate. Factors associated with the prescription of reduced-dose of NOACs included older age, renal disease, bleeding risk and the concomitant use of drugs predisposing to bleeding. Given that treatment with reduced doses is dependent largely on clinical and demographic characteristics, the high prevalence of older patients observed to be on adjusted doses in our unselected nationwide population is not unexpected^6^. Indeed, these percentages are much higher than the frequency reported in key trials^11–14^ (dabigatran 50%, rivaroxaban 21%, apixaban 5% and edoxaban 25%) and in real-world data from observational studies and registries^18–20^. In the ORBIT-AF II Registry, only 16% of patients were prescribed reduced dose, and among all NOACs the highest percentage was for apixaban, the NOAC with the most restrictive criteria dose-adjustment. Nevertheless, these data may represent an optimistic assessment of doses due to the potential biases of participation in an AF registry^18,19^. A similar percentage (18%) was reported on a large US administrative database^20^.

As a matter of fact, our patients on reduced dose represent a complex population as shown by the 37% of patients aged 85 years or more, more than one third with heart failure, with high risk of stroke (CHA_2_DS_2_-VASc ≥ 4 in 88%) and bleeding (HAS-BLED ≥ 3 in 51%), previous bleeding or conditions predisposing to bleeding.

The presence of renal insufficiency justifies a dose-reduction to maintain serum concentration comparable to that of patients with normal renal function treated with standard doses^21^. Surprisingly, prescribers identified only 10% of patients with renal disease as evidenced by the data. This prevalence is probably underestimated, since it was not required by the Registry to specify the individual renal function, but only to classify the patient as having the disease, and thus taking into account the possibility that the impaired renal function could not be recognized or indicated by prescribing physicians in a consistent proportion of patients^22^.

Except for age, the characteristics of patients on reduced dose of NOACs in AIFA Registry were not remarkably different from those reported by others^19,21^, which provides the opportunity to initiate NOAC treatment for patients previously excluded from treatment with VKA because of elevated risks of complications. In the study of Chao et al.^23^, the treatment with NOACs of more than 11,000 patients 90 years old or more was confirmed as effective and safe in comparison with VKA. In AIFA Registry, about 49,000 patients were 90 years old or more, and it would be indeed relevant to confirm both the efficacy and safety of NOACs in the Italian region.

To our knowledge, there is scant information available in the literature on the historical trend of prescription of different dose of NOACs, in particular in a large, unselected, nationwide registry. In a contemporary Japanese registry^24^ nearly 40% of patients on NOACs were on reduced dose and this percentage slightly increased in the 5-year study observation period. Conversely in AIFA Registry, there was a clear trend towards a reduction of the proportion of patients treated with reduced dose overtime. This was evident for dabigatran and rivaroxaban, as well as apixaban, considering the increasing proportion of the very elderly treated with this drug and the progressive decrease of prescription of its adjusted dose in patients aged 85 years or more.

Use of lower doses has been reported inappropriate, that is, inconsistent with drug labeling, in up to half of the patients in some papers^19,24,25^, while in a lower proportion in others^26,27^.

Inappropriate dosing, and especially under-dosing, has been associated in turn with unfavorable outcomes. The FDA previously expressed concerns regarding off-label under-dosing of anticoagulants^28^. FDA criteria^29,30^ are very similar to ESC/EHRA ones^1,3^, with the only difference regarding edoxaban, where American guidelines require reduction of the dose only in instances of impaired renal function, whereas European ones also include a low body weight and the use of Gp inhibitors.

In AIFA Registry, among patients with the criteria to be treated with standard doses, 30 to 40% were treated with reduced dose with only a minority of them showing some other characteristics predisposing to bleeding which may explain the dose choice. (Fig. 4).

Moreover, apixaban was the NOAC with the highest difference in the proportion of reduced-dose reported in AIFA Registry (41%) and in the key trial (only 5%)^13^. Italian patients receiving apixaban low dose, in comparison with those on standard dose, were substantially older (48% vs 9% of patients ≥ 85), mostly women, with more frequent comorbidities (including renal disease), and more likely to have a history of stroke and bleeding. Thus, the choice was roughly consistent with the criteria for dose reduction. While the large majority of elderly patients (≥ 80 years) and impaired renal function were appropriately on low dose of apixaban, a proportion of patients much higher than previously reported^31,32^ were on low dose of apixaban in spite of being younger than 80 or classified without renal disease. In the study of Li et al.^31^, 16.1% of patients treated with apixaban were on reduced dose. These patients were very similar to ours, with the exception of a higher frequency of renal disease and HAS-BLED score ≥ 3. Compared with warfarin, low dose apixaban was associated with a 37% lower risk of stroke/SE and non-significant trend toward a lower risk of hemorrhagic stroke.

Conversely, one third of patients on low dose reported by Coleman et al.^32^, in comparison with ours^6^, were younger and with lower CHA_2_DS_2_-VASc, but with similar renal disease and risk of bleeding. Remarkably, patients on reduced dose in this study showed a trend towards a higher risk of ischemic stroke versus VKA users.

Our data did not offer a clear explanation to understand the dose choice in about 25% of patients treated with low dose of apixaban. This is similar to the results of others^26^ reporting a similar proportion of potential under-dosing of apixaban. It is possible that also in AIFA Registry the majority of patients inappropriately prescribed a reduced dose of apixaban met only one dose-reduction criteria. The lack of identification of patients with renal impairment in the Registry^22^, as far as the proportion of those with body weight ≤ 60 kg, may justify the choice in a subgroup of the other patients.

Similar reasons may partially explain the proportion of patients treated with reduced dose of rivaroxaban and edoxaban, much higher than in other experiences^26,27^.

Dabigatran is the only drug formally tested and approved in both doses in Europe. Thus, within the approved criteria (GFR > 30 ml/min), the prescription of reduced dose should not be considered in any case as inappropriate. Nevertheless, the FDA previously expressed specific concern regarding off-label under-dosing of dabigatran^28^ and Lip et al.^7^ suggested to prescribe reduced dose in patients of 80 years or more, or in those with high bleeding risk, while no dose-adjustment is required for GFR 30–50 ml/min. Applying these criteria, 32% of patients on dabigatran were potentially undertreated (< 80 years AND HAS-BLED < 3).

A critical interaction mechanism for all NOACs consists of competitive inhibition of P-gp transporter and CYP3A4, associated with an increase in plasma levels. Many antiarrhythmic drugs used in AF patients are P-gp inhibitors (e.g., verapamil, diltiazem, dronedarone, amiodarone, and quinidine), others (some antibiotics, antiviral drugs, fungistatic, anticancer and antiepileptic drugs) are strong inhibitors of CYP3A4. In case of co-administration of these drugs, clinical guidelines recommend prescribing reduced doses of NOACs or to avoid the concomitant use^3^.

In the AIFA Registry, a higher proportion of prescription of drugs predisposing to bleeding (mainly antiplatelets) was present in the reduced dose group of patients (19% vs 14%) and may explain another small part of prescription of low dose NOACs. Consistently, the prescription of medication predisposing to bleeding, including the temporary prescription of single or dual antiplatelet therapy after acute coronary syndrome, was associated with switching from reduced to standard dose at renewal of prescription.

The drug with the highest odds of dose-reduction at the second prescription was dabigatran. Conversely, these data suggest that a sizeable proportion of patients are receiving reduced-dose of NOACs based on other characteristics (including personal evaluation of prescribers) and not on either guideline recommended labeling or classic markers of bleeding risk. The underlying risks of the cohort treated with low dose NOACs suggest potential intentional deviation from approved doses. In general, it is possible that prescribers often under-dose medications in an effort to “do no harm.” For example, patients who were under-dosed had significantly higher risk as expressed by older age, renal disease and bleeding risk than those given doses on-label. Physicians may adapt the doses of these drugs according to the specific patient’s risk, even if data from clinical trials and subgroup analyses demonstrated a favorable risk–benefit profile whatever the risk^11–14,33^.

The risk of under-dosing was the reason why the FDA did not approve the lower dose of dabigatran, thereby preventing the physicians to use the lower dose in order to minimize bleeding, at the expense of the increase of thromboembolic events^28^. Our data suggest such concerns may have been valid. A significant proportion of patients received off-label dosing, and such dosing with these agents may have increased the risk of worse outcomes.

### Strengths and limitations

Our study is the largest ever published on NOAC dosing, including all patients treated in a large European country. Furthermore, the cohort includes large proportion of older participants and has the highest percentage of patients treated with reduced dose NOACs in a nationwide unselected registry.

Nevertheless, some limitations do exist. The peculiarity of Italian web-based registries, consisting of a mandatory therapeutic plan required for strict control of prescriptions and the absence of a supervised monitoring of data entry and audit visits, could have negatively influenced the quality and accuracy of clinical data.

Moreover, we did not have some clinical information at baseline (such as weight, detailed renal or hepatic impairment, exact alcohol consumption, potential drug interactions) and it is likely that some other unmeasured residual confounding factors were still present. As pointed out by others^18^ one of the main critical point was the identification of patients with renal impairment. Renal function is evaluated by creatinine for apixaban, and by estimated CrCl for the other NOACs, as calculated by the Cockroft-Gault formula and routinely according to the MDRD or CKD EPI formula in clinical practice. The well-described differences between these methods may justify different choice of dosages, in particular for border cases. Moreover, in absence of the timely information, the record of diagnosis “kidney disease” probably underestimated the true proportion of patients with renal impairment.

Therefore, we might have overestimated the potentially inappropriate reduced dose of NOACs. The actual lack of linkage with the national outcome databases prevented us from collecting data on effectiveness and safety of NOACs in Italian population. These critical issues require further investigation.

## Conclusions

This study portrays the real picture of the entire Italian NOACs prescription, focusing on dosage choice. Evidence based on real-world data shows a high frequency of low dose prescriptions of NOACs in patients with AF. Older age, renal disease, bleeding risk and the concomitant use of drugs predisposing to bleeding, determined the choice of reduced dose. These data may suggest a potential under-treatment of AF patients, but further evaluation is warranted. The physician’s apprehension regarding excessive bleeding must be balanced with a consideration for an increased risk of embolic events, particularly in patients with a significant risk profile.

Further analyses are needed to analyze the effectiveness and safety of NOACs in Italy. Nonetheless, future studies on reduced dose NOAC versus warfarin are still warranted and should preferably analyze effectiveness and safety outcomes in respect to label adherence.

## Supplementary Information

Supplementary Tables.
