# Supplementary material for: Starting dose and dose adjustment of non-vitamin K antagonist oral anticoagulation agents in a nationwide cohort of patients with atrial fibrillation
Source: Sci Rep. 2021 Oct 19;11:20689. doi: 10.1038/s41598-021-99818-4 (PMC8526656; doi:10.1038/s41598-021-99818-4)
Supplement: Supplementary file 1 — Supplementary Tables. [file 41598_2021_99818_MOESM1_ESM.docx]

**Supplementary Table S1a:** Baseline characteristics of the cohort treated with standard dose of NOACs at first prescription in AIFA Registry

| Characteristics | Dabigatran | Rivaroxaban | Apixaban | Edoxaban |
| --- | --- | --- | --- | --- |
| N° treatments | 96926 (39.68%) | 192654 (62.60%) | 168535 (59.09%) | 49433 (54.79%) |
| Sex |  |  |  |  |
| Female | 37164 (38.34%) | 85115 (44.18%) | 78895 (46.81%) | 22092 (44.69%) |
| Male | 59762 (61.66%) | 107539 (55.82%) | 89640 (53.19%) | 27341 (55.31%) |
| Median age (range) |  |  |  |  |
| Age < 65 | 24823 (25.61%) | 34093 (17.7%) | 21345 (12.67%) | 7431 (15.03%) |
| Age ≥ 65 & <75 | 47891 (49.41%) | 67952 (35.27%) | 57607 (34.18%) | 17131 (34.65%) |
| Age ≥ 75 & <85 | 23514 (24.26%) | 76234 (39.57%) | 74404 (44.15%) | 20566 (41.6%) |
| Age ≥ 85 | 698 (0.72%) | 14375 (7.46%) | 15179 (9.01%) | 4305 (8.71%) |
| CHA₂DS₂-VASc Score 0 | 1387 (1.43%) | 2988 (1.55%) | 1022 (0.61%) | 394 (0.8%) |
| CHA₂DS₂-VASc Score 1 | 9396 (9.69%) | 13332 (6.92%) | 8081 (4.79%) | 3018 (6.11%) |
| CHA₂DS₂-VASc Score 2 | 22462 (23.17%) | 31889 (16.55%) | 24243 (14.38%) | 8465 (17.12%) |
| CHA₂DS₂-VASc Score 3 | 27449 (28.32%) | 48892 (25.38%) | 42184 (25.03%) | 13320 (26.95%) |
| CHA₂DS₂-VASc Score 4 | 19768 (20.39%) | 47890 (24.86%) | 44140 (26.19%) | 12704 (25.7%) |
| CHA₂DS₂-VASc Score 5 | 10180 (10.5%) | 27151 (14.09%) | 26924 (15.98%) | 6804 (13.76%) |
| CHA₂DS₂-VASc Score 6+ | 6284 (6.48%) | 20512 (10.65%) | 21941 (13.02%) | 4728 (9.56%) |
| HAS-BLED Score 0 | 4419 (4.56%) | 7132 (3.7%) | 3624 (2.15%) | 1379 (2.79%) |
| HAS-BLED Score 1 | 21423 (22.1%) | 35607 (18.48%) | 26947 (15.99%) | 9942 (20.11%) |
| HAS-BLED Score 2 | 37960 (39.16%) | 81274 (42.19%) | 70911 (42.07%) | 22673 (45.87%) |
| HAS-BLED Score 3 | 22456 (23.17%) | 46521 (24.15%) | 44225 (26.24%) | 10555 (21.35%) |
| HAS-BLED Score 4+ | 10668 (11.01%) | 22120 (11.48%) | 22828 (13.54%) | 4884 (9.88%) |
| Diabetes history | 18817 (19.41%) | 36488 (18.94%) | 33597 (19.93%) | 9130 (18.47%) |
| Hypertension history | 82846 (85.47%) | 164120 (85.19%) | 145076 (86.08%) | 42258 (85.49%) |
| Stroke/TIA/Thrombo-embolism history | 15813 (16.31%) | 27803 (14.43%) | 29757 (17.66%) | 6339 (12.82%) |
| Vascular disease history | 20905 (21.57%) | 47590 (24.7%) | 42229 (25.06%) | 11460 (23.18%) |
| CHF history | 19348 (19.96%) | 45808 (23.78%) | 39947 (23.7%) | 10694 (21.63%) |
| Alcohol use | 5865 (6.05%) | 9951 (5.17%) | 9291 (5.51%) | 3124 (6.32%) |
| Liver disease | 846 (0.87%) | 1387 (0.72%) | 1342 (0.8%) | 314 (0.64%) |
| Renal disease | 1008 (1.04%) | 2356 (1.22%) | 4117 (2.44%) | 641 (1.3%) |
| Prior major bleeding or predisposition to bleeding | 6402 (6.61%) | 13909 (7.22%) | 17341 (10.29%) | 3665 (7.41%) |
| Labile INR | 23790 (24.54%) | 42847 (22.24%) | 34071 (20.22%) | 7641 (15.46%) |
| Prior AVK treatment | 34758 (35.86%) | 58998 (30.62%) | 47752 (28.33%) | 11130 (22.52%) |
| Medication usage predisposing to bleeding | 13201 (13.62%) | 28129 (14.6%) | 25633 (15.21%) | 6258 (12.66%) |
| Prior NOAC treatment (switch) | 2030 (2.09%) | 9026 (4.69%) | 13279 (7.88%) | 4185 (8.47%) |

**Table S1b:** Baseline characteristics of the cohort treated with reduced dose of NOACs at first prescription in AIFA Registry

| Characteristics | Dabigatran | Rivaroxaban | Apixaban | Edoxaban |
| --- | --- | --- | --- | --- |
| N° treatments | 147355 (60.32%) | 115119 (37.40%) | 116705 (40.91%) | 40796 (45.21%) |
| Sex |  |  |  |  |
| Female | 76318 (51.79%) | 66533 (57.79%) | 71111 (60.93%) | 25549 (62.63%) |
| Male | 71037 (48.21%) | 48586 (42.21%) | 45594 (39.07%) | 15247 (37.37%) |
| Age |  |  |  |  |
| < 65 | 5444 (3.69%) | 2319 (2.01%) | 1353 (1.16%) | 705 (1.73%) |
| ≥ 65 & <75 | 22394 (15.2%) | 11979 (10.41%) | 7676 (6.58%) | 3894 (9.55%) |
| ≥ 75 & <85 | 82992 (56.32%) | 56751 (49.3%) | 51761 (44.35%) | 17764 (43.54%) |
| ≥ 85 | 36525 (24.79%) | 44070 (38.28%) | 55915 (47.91%) | 18433 (45.18%) |
| CHA₂DS₂-VASc Score 0 | 290 (0.2%) | 72 (0.06%) | 58 (0.05%) | 22 (0.05%) |
| CHA₂DS₂-VASc Score 1 | 1688 (1.15%) | 613 (0.53%) | 418 (0.36%) | 235 (0.58%) |
| CHA₂DS₂-VASc Score 2 | 8759 (5.94%) | 4351 (3.78%) | 3313 (2.84%) | 1639 (4.02%) |
| CHA₂DS₂-VASc Score 3 | 29141 (19.78%) | 18361 (15.95%) | 16228 (13.91%) | 6674 (16.36%) |
| CHA₂DS₂-VASc Score 4 | 45678 (31%) | 36392 (31.61%) | 35787 (30.66%) | 13341 (32.7%) |
| CHA₂DS₂-VASc Score 5 | 32224 (21.87%) | 28679 (24.91%) | 30101 (25.79%) | 10012 (24.54%) |
| CHA₂DS₂-VASc Score 6+ | 29575 (20.07%) | 26651 (23.15%) | 30800 (26.39%) | 8873 (21.75%) |
| HAS-BLED Score 0 | 796 (0.54%) | 254 (0.22%) | 161 (0.14%) | 145 (0.36%) |
| HAS-BLED Score 1 | 15007 (10.18%) | 10126 (8.8%) | 9902 (8.48%) | 4617 (11.32%) |
| HAS-BLED Score 2 | 56164 (38.11%) | 46275 (40.2%) | 45184 (38.72%) | 18189 (44.59%) |
| HAS-BLED Score 3 | 46529 (31.58%) | 34973 (30.38%) | 36002 (30.85%) | 10930 (26.79%) |
| HAS-BLED Score 4+ | 28859 (19.58%) | 23491 (20.41%) | 25456 (21.81%) | 6915 (16.95%) |
| Diabetes history | 29717 (20.17%) | 23217 (20.17%) | 23413 (20.06%) | 7707 (18.89%) |
| Hypertension history | 128882 (87.46%) | 101096 (87.82%) | 100391 (86.02%) | 34958 (85.69%) |
| Stroke/TIA/Thromboembolism history | 29285 (19.87%) | 21669 (18.82%) | 25005 (21.43%) | 6953 (17.04%) |
| Vascular disease history | 44964 (30.51%) | 35598 (30.92%) | 36481 (31.26%) | 11440 (28.04%) |
| CHF history | 43369 (29.43%) | 43971 (38.2%) | 46225 (39.61%) | 14832 (36.36%) |
| Alcohol use | 7195 (4.88%) | 4410 (3.83%) | 4110 (3.52%) | 1810 (4.44%) |
| Liver disease | 1771 (1.2%) | 1199 (1.04%) | 1477 (1.27%) | 433 (1.06%) |
| Renal disease | 6025 (4.09%) | 13898 (12.07%) | 17512 (15.01%) | 5485 (13.44%) |
| Prior major bleeding or predisposition to bleeding | 19811 (13.44%) | 14843 (12.89%) | 21552 (18.47%) | 5674 (13.91%) |
| Labile INR | 37661 (25.56%) | 24423 (21.22%) | 20108 (17.23%) | 5605 (13.74%) |
| Prior AVK treatment | 52857 (35.87%) | 32387 (28.13%) | 26742 (22.91%) | 7838 (19.21%) |
| Medication usage predisposing to bleeding | 32235 (21.88%) | 21879 (19.01%) | 20085 (17.21%) | 5821 (14.27%) |
| Prior NOAC treatment (switch) | 4458 (3.03%) | 8225 (7.14%) | 13608 (11.66%) | 5919 (14.51%) |

**Table S2:** Baseline characteristics of patients for each prescribed NOAC during the study period in AIFA Registry.

| **Characteristics** | **2013** | **2014** | **2015** | **2016** | **2017** | **2018** |
| --- | --- | --- | --- | --- | --- | --- |
| **Apixaban** |  |  |  |  |  |  |
| **Age 65-** | - | 6,17 | 8,84 | 8,35 | 7,68 | 7,93 |
| **Age 65 & 74** | - | 26,74 | 24,33 | 22,43 | 21,74 | 21,33 |
| **Age 75 & 84** | - | 46,20 | 44,56 | 44,33 | 43,85 | 43,20 |
| **Age 85+** | - | 20,89 | 22,27 | 24,89 | 26,73 | 27,54 |
| **Renal disease** | - | 9,36 | 7,60 | 7,18 | 7,02 | 7,72 |
| **Liver disease** | - | 1,14 | 1,02 | 1,03 | 0,95 | 0,87 |
| **Medication usage predisposing to bleeding** | - | 19,84 | 18,36 | 16,51 | 14,39 | 13,13 |
| **Prior major bleeding or predisposition to bleeding** | - | 16,31 | 14,96 | 13,50 | 12,71 | 12,20 |
| **Edoxaban** |  |  |  |  |  |  |
| **Age 65-** | - | - | - | 9,54 | 9,11 | 8,87 |
| **Age 65 & 74** | - | - | - | 25,68 | 23,99 | 22,37 |
| **Age 75 & 84** | - | - | - | 43,39 | 42,37 | 42,49 |
| **Age 85+** | - | - | - | 21,39 | 24,53 | 26,28 |
| **Renal disease** | - | - | - | 7,21 | 6,55 | 6,97 |
| **Liver disease** | - | - | - | 0,77 | 0,80 | 0,86 |
| **Medication usage predisposing to bleeding** | - | - | - | 14,41 | 14,10 | 12,57 |
| **Prior major bleeding or predisposition to bleeding** | - | - | - | 11,79 | 10,56 | 9,99 |
| **Dabigatran** |  |  |  |  |  |  |
| **Age 65-** | 9,44 | 12,46 | 13,00 | 13,45 | 12,66 | 13,32 |
| **Age 65 & 74** | 29,75 | 30,36 | 29,23 | 29,11 | 27,32 | 26,75 |
| **Age 75 & 84** | 45,98 | 43,54 | 43,48 | 42,88 | 43,28 | 42,45 |
| **Age 85+** | 14,82 | 13,64 | 14,30 | 14,57 | 16,74 | 17,48 |
| **Renal disease** | 3,88 | 2,91 | 2,56 | 2,67 | 2,45 | 2,85 |
| **Liver disease** | 0,97 | 1,09 | 1,07 | 1,17 | 1,02 | 1,11 |
| **Medication usage predisposing to bleeding** | 19,30 | 19,69 | 18,76 | 17,08 | 17,41 | 19,44 |
| **Prior major bleeding or predisposition to bleeding** | 13,46 | 11,82 | 9,93 | 9,63 | 9,63 | 9,89 |
| **Rivaroxaban** |  |  |  |  |  |  |
| **Age 65-** | 7,43 | 9,21 | 10,64 | 11,59 | 13,44 | 14,19 |
| **Age 65 & 74** | 24,98 | 25,19 | 25,88 | 25,86 | 26,46 | 26,39 |
| **Age 75 & 84** | 47,53 | 46,28 | 44,67 | 43,22 | 41,45 | 40,67 |
| **Age 85+** | 20,06 | 19,31 | 18,81 | 19,33 | 18,65 | 18,74 |
| **Renal disease** | 8,96 | 7,09 | 5,13 | 4,63 | 4,64 | 4,94 |
| **Liver disease** | 1,22 | 1,07 | 0,91 | 0,86 | 0,74 | 0,64 |
| **Medication usage predisposing to bleeding** | 20,90 | 19,10 | 17,46 | 15,88 | 15,08 | 13,90 |
| **Prior major bleeding or predisposition to bleeding** | 14,27 | 12,34 | 10,33 | 8,93 | 8,04 | 7,25 |
